# Paired analysis of tree ring width and carbon isotopes indicates when controls on tropical tree growth change from light to water limitations

**DOI:** 10.1093/treephys/tpab142

**Published:** 2021-10-29

**Authors:** Roel Brienen, Gerhard Helle, Thijs Pons, Arnoud Boom, Manuel Gloor, Peter Groenendijk, Santiago Clerici, Melanie Leng, Christopher Jones

**Affiliations:** School of Geography, University of Leeds, Leeds LS2 9JT, UK; GFZ—German Research Centre for Geosciences, Section 4.3 Climate Dynamics and Landscape Evolution, 14473 Potsdam, Germany; Plant Ecophysiology, Institute of Environmental Biology, Utrecht University, 3512 Utrecht, The Netherlands; School of Geography, University of Leicester, Leicester LE1 7RH, UK; School of Geography, University of Leeds, Leeds LS2 9JT, UK; Department of Plant Biology, Institute of Biology, PO Box: 6109, University of Campinas, UNICAMP, Campinas 13083-970, Brazil; Ecology and Biodiversity, Institute of Environmental Biology, Utrecht University, 3584 Utrecht, The Netherlands; School of Geography, University of Leeds, Leeds LS2 9JT, UK; National Environmental Isotope Facility, British Geological Survey, Nottingham NG12 5GG, UK; School of Geography, University of Leeds, Leeds LS2 9JT, UK

**Keywords:** gap dynamics, growth release, suppression, tree rings, tropical forest

## Abstract

Light and water availability are likely to vary over the lifespan of closed-canopy forest trees, with understory trees experiencing greater limitations to growth by light and canopy trees greater limitation due to drought. As drought and shade have opposing effects on isotope discrimination (Δ^13^C), paired measurement of ring width and Δ^13^C can potentially be used to differentiate between water and light limitations on tree growth. We tested this approach for *Cedrela* trees from three tropical forests in Bolivia and Mexico that differ in rainfall and canopy structure. Using lifetime ring width and Δ^13^C data for trees of up to and over 200 years old, we assessed how controls on tree growth changed from understory to the canopy. Growth and Δ^13^C are mostly anti-correlated in the understory, but this anti-correlation disappeared or weakened when trees reached the canopy, especially at the wettest site. This indicates that understory growth variation is controlled by photosynthetic carbon assimilation due to variation in light levels. Once trees reached the canopy, inter-annual variation in growth and Δ^13^C at one of the dry sites showed positive correlations, indicating that inter-annual variation in growth is driven by variation in water stress affecting stomatal conductance. Paired analysis of ring widths and carbon isotopes provides significant insight in what environmental factors control growth over a tree’s life; strong light limitations for understory trees in closed-canopy moist forests switched to drought stress for (sub)canopy trees in dry forests. We show that combined isotope and ring width measurements can significantly improve our insights in tree functioning and be used to disentangle limitations due to shade from those due to drought.

## Introduction

Besides CO_2_, all trees require light and water for growth and survival. The relative supply of these resources is often inversely related over a tree’s life time. For example, small understory trees are often severely light-limited especially in tall closed-canopy tropical forests (Kira et al. 1969, Turner 2002, Poorter et al. 2005), while canopy trees face difficulties keeping their crowns well-watered (Ryan et al. 2006, McDowell and Allen 2015). The availability of these resources similarly varies between forests, with greater competition for light in wet forests and greater drought stress in dry forests. Disentangling the relative strengths of light versus water controls on tree photosynthesis, and how they impact growth throughout a tree’s life, is important for understanding forest dynamics and differences in tree functioning under different climatic and environmental conditions.

Tropical forest trees in the understory often receive only a fraction (1–2%) of the light levels of canopy trees (Chazdon et al. 1984, Montgomery and Chazdon 2001). This lack of light strongly limits the growth of tropical forest seedlings and saplings, which may remain supressed for several decades or longer and require growth releases for trees to get to the canopy (Clark and Clark 2001, Baker and Bunyavejchewin 2006, Brienen et al. 2010*a*). Once trees reach the canopy, competition for light with surrounding trees is reduced, and water demand increases due to an increase in irradiance and leaf internal to atmospheric vapor pressure deficit (VPD; Kira and Yoda 1989). At the same time, the longer pathlength from soil to tree crown will increase the resistance of water transport for tall trees (Ryan and Yoder 1997). Despite mechanisms to cope with increased resistance for water transport, such as greater investment in roots (Dawson 1996, Slot et al. 2012, Brum et al. 2019), water stress increases as trees increase in height (Koch et al. 2004, Ryan et al. 2006). These lifetime changes when growing from the understory to the upper canopy are known to affect tree’s leaf morphology and physiology (Rijkers et al. 2000, Cavaleri et al. 2010, McDowell et al. 2011, Steppe et al. 2011, Houter and Pons 2012), tree hydraulics (Olson et al. 2018) and patterns of tree growth (Clark and Clark 1992, 2001), reproduction (Thomas 2011) and mortality (Bennett et al. 2015, Johnson et al. 2018). However, there is still a lack of detailed insight into how these changes in light and water availability over a trees’ life may limit growth throughout tree ontogeny in different forest types, especially for tropical trees.

Tree ring studies have improved our understanding of controls on tropical tree growth. Climate growth analysis in tropical trees showed that growth can be limited by the amount of rainfall (Vlam et al. 2014, Granato-Souza et al. 2019) with stronger controls of rainfall in dry forests (Brienen et al. 2010*b*, López and Villalba 2011, Mendivelso et al. 2014). In forests with higher water availability and denser canopies, competition is more important than precipitation, especially when trees are small (Baker and Bunyavejchewin 2006, Brienen et al. 2010*a*). Temporal analysis of ring widths indicates that growth in moist forests is more strongly controlled by variation in light due to canopy gap dynamics, resulting in longer periods of suppressions and more growth releases compared with drier forests (Brienen et al. 2010*a*). These results indicate that the effects of light and water on growth are likely to vary between different life stages and forest types. However, a lack of historical records of light levels at the time of ring formation means that analysis of temporal variation in tree rings alone does not provide conclusive evidence of to what degree growth is controlled by variation in understory light levels or arises from other controls. One potentially useful tool to obtain insights in the effects of light and water availability on tree growth is the analysis of stable carbon isotopes in tree rings (δ^13^C_tr_). Carbon isotopes provide information on the relative strengths of limits to carbon assimilation versus stomatal conductance on photosynthesis (Farquhar and Richards 1984, Barbour and Farquhar 2000, Aranda et al. 2007).

From δ^13^C_tr_ and historical records of δ^13^C_air_, the plant-to-air isotope discrimination (Δ^13^C_tr_) can be calculated as follows:

(1) \begin{equation*} \Delta^{13} {\rm{C}}_{\rm tr} = (\delta^{13} {\rm{C}}_{\rm air} - \delta^{13} {\rm{C}}_{\rm tr})/(1 + \delta^{13} {\rm{C}}_{\rm tr}/1000).\end{equation*}

Variation in Δ^13^C_tr_ arises from changes in the ratio of leaf intercellular to atmospheric [CO_2_], *C*_i_/*C*_a_ during photosynthesis. The simplest model relating these two metrics is the linear or reduced model of Farquhar et al. (1982), where Δ^13^C_tr_ = *a* + (*b* − *a*) ^*^ (*c*_i_/*c*_a_), with *a* (4.4‰) referring to discrimination due to slower diffusion of ^13^CO_2_ compared with ^12^CO_2_ through the stomata, and *b* (27‰) to discrimination by the CO_2_-fixing enzyme Rubisco. According to this model, for a constant atmospheric CO_2_ level (*C*_a_), a change in Δ^13^C_tr_ thus reflects changes in *c*_i_, which can result from a change in uptake of CO_2_ through assimilation (*A*) and/or a change in CO_2_ supply regulated by stomatal conductance (*g*_s_). For example, if photosynthesis rate (*A*) is limited by carboxylation as a result of light limitation (and not by CO_2_ supply), *C*_i_ and therefore Δ^13^C_tr_ increases (Carelli et al. 1999, Coste et al. 2010). Limits on photosynthesis due to low CO_2_ supply as a result of low stomatal conductance (*g*_s_) caused by, e.g., limitations in water supply, result in a decrease in *c*_i_ and therefore Δ^13^C_tr_ (Farquhar et al. 1989). Combined analysis of growth (i.e., ring width) and Δ^13^C_tr_ thus may enable a determination of the degree to which growth is limited by light or water availability. Growth increases associated with lower Δ^13^C_tr_ (i.e., anti-correlations of Δ^13^C_tr_ and growth) indicate that growth is predominantly controlled by variation in light availability, while positive correlations between growth and Δ^13^C_tr_ indicate controls of stomatal conductance and water availability on growth (van der Sleen et al. 2014, Giuggiola et al. 2016). Some studies have previously used this approach to disentangle drought from light effects (Saurer et al. 1997, van der Sleen et al. 2014, Voelker et al. 2014, Giuggiola et al. 2016), but no study has used this to evaluate how these controls may change over a tree’s life.

**Figure 1.**
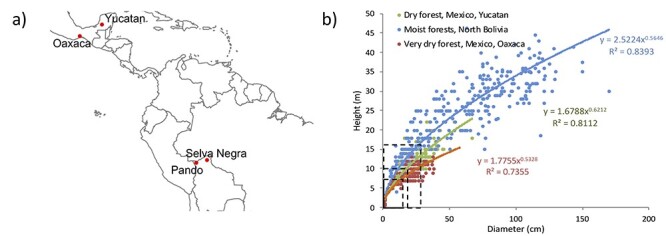
Site locations (a) and allometric diameter–height relationships (b) for all sites. Note that data for the two locations in northern Bolivia were treated as one single site. Lines in (b) indicate the assumed tree height and diameter thresholds at which trees reach the lower forest canopy (see Table 1).

Previous analysis of temporal growth patterns of trees toward the canopy identified longer periods of suppressions and more growth releases for trees growing at a moist tropical forest with high canopy compared with a more open, low stature dry forest (Brienen et al. 2010*a*). These differences are most likely driven by greater spatial and temporal variation in trees’ light environment, but growth rate analysis by itself does not allow drivers of variation to be distinguished. By combining tree ring width and δ^13^C_tr_ data, we here test whether this approach is indeed suitable to indicate light versus water limitation, and study whether limitations on growth change throughout a tree’s lifetime or vary between sites. The study comprises tropical *Cedrela* trees from three sites differing in annual rainfall, dry season length, stand density and canopy height (Figure 1, Table 1). These trees form distinct proven annual rings and reach ages of up to 300 years (Brienen et al. 2010*a*).

**Table 1:** Characteristics of the forests and the *Cedrela* trees at the three study sites.

Site	Forest type	Forest height	Soil type	Rainfall	*Cedrela*			
					Max tree height	Max diameter	Height lower canopy^1^	Diameter lower canopy^1^
Northern Bolivia	Tropical semi-deciduous	30–35 m	Xanthic ferrasols	1750 mm	46 m	180 cm	~17 m	~30 cm
Yucatan	Tropical dry	~15 m	Karstic	1100 mm	22 m	52 cm	~10 m	~20 cm
Oaxaca	Tropical dry	~10 m	Karstic	900 mm	13 m	38.5 cm	~7.5 m	~15 cm

^1^Defined as tree height corresponding to about two-thirds of the total canopy height.

Our hypothesis is that trees are generally more light limited in the understory and thus show negative correlations between growth and Δ^13^C when small, but that these correlations disappear when reaching the canopy. We further anticipate that the relative strength of light and water limitation varies between sites, with (i) a stronger light limitation of growth in the wetter sites resulting in anti-correlations between growth and Δ^13^C_tr_, and (ii) a stronger water availability limitation on growth at the driest site resulting in positive correlations between growth and Δ^13^C_tr_. We test these hypotheses by analyzing temporal co-variation in growth and Δ^13^C_tr_ over a tree’s life from understory to canopy stages and compare these patterns for the different sites. We further assess the relative effects of tree height, light and atmospheric CO_2_ on Δ^13^C_tr_ (cf., McDowell et al. 2011, Brienen et al. 2017, Vadeboncoeur et al. 2020), as these are fundamental to the interpretation of our results. For example, changes in light as trees grow to the canopy are accompanied by changes in tree height, as well as changes in atmospheric CO_2_ concentration due to CO_2_ emissions.

## Materials and methods

### Study sites and species

This study is based on data from four locations (Table 1, Figure 1). Two sampling locations are located in northern Bolivia in the department of Pando, one 50 km south of Cobija (Purissima, 11°24′S, 68°43′W) and one north of Riberalta (Selva Negra, 10°5′S, 66°18′W). These sample locations (cf., 350 km apart) are treated as one site as they share the same climate and vegetation. The annual precipitation is ~1750 mm with a distinct, dry season (<50 mm per month) of 3 months from June to August. The vegetation consists of semi-deciduous, moist tropical forests with a maximum canopy height of 30–35 m. The other two sites are in Mexico, in the state of Campeche on the Yucatan Peninsula (Ejido Pich, 19°03′N, 90°00′W), and in the state of Oaxaca on the Pacific slope of the Isthmus of Tehuantepec, close to Nizanda (16°39′N, 95°00′W). Both sites are much drier than those in northern Bolivia. The site in Yucatan receives ~1100 mm annual precipitation and has a 5-month long dry season (December–April), and the vegetation consists of tropical dry forest mostly on hilly terrain with karstic soils and very good drainage. Average canopy height at this site is ~15 m and the forest structure is open. The site in Oaxaca receives ~ 930 mm rainfall with a 7-month long dry season (November–May) and consists of tropical dry forest on steep slopes and karstic soils. Canopy height varies between 10 and 15 m and the forest structure is very open. All sites are old growth forests, although two of the sites (Selva Negra and Yucatan) have experienced selective logging in the past of less than one tree per hectare.

The sampled tree species are *Cedrela odorata* L. in Bolivia and Yucatan and *Cedrela salvadorensis* Standl. (Meliaceae) in Oaxaca. Both are deciduous species that lose their leaves during the dry season and form distinct annual rings, marked by terminal parenchyma bands often in conjunction with variation in vessel density and size (Worbes 1999, Baker et al. 2017). *Cedrela odorata* is a relatively light demanding canopy tree that can survive for relatively long periods at low growth and reach ages of over 300 years (Brienen et al. 2010*a*). In moist forest, the species reached heights of up to 45 m and diameters of 200 cm. *Cedrela salvadorensis* performs best as seedlings in intermediate light conditions (Guzmán-Q et al. 2016). The species reaches heights of up to 15 m, diameters of 50 cm and ages of ~120 years (Groenendijk 2010).

### Sample collections and field measurements

Samples used in this study consisted of a mix of large stem disks, disk fragments and increment cores of trees. Samples were taken in 2001 (Bolivia, Purissima), 2007 (Yucatan and Oaxaca) and 2011 (Bolivia, Selva Negra) from trees ranging in size from 50-cm tall seedlings to canopy trees. At all three sites, tree ring chronologies were build using standard ring width cross-dating (Brienen and Zuidema 2005, Brienen et al. 2010*a*), complemented with oxygen isotope chronologies (Brienen et al. 2012, Baker et al. 2015). For the sites in Bolivia, we collected over 100 stem discs from large trees (>60 cm in diameter at breast height, DBH) and 55 small discs from seedlings and saplings, and increment cores (2–3 radii) from >150 trees. In Yucatan, we collected 10 discs from large trees and cores from 70 trees (2–3 radii), and in Oaxaca, we collected 6 discs and increment cores (2–3 radii) from 70 trees. For each tree, we measured its DBH, estimated tree height and assessed light availability using the modified Crown Illumination Index (CII) of Dawkins (Clark and Clark 1992) varying from 1 (no direct lateral or overhead light) to 5 (full overhead and lateral, direct light). The CII values of trees at the Bolivian and the Yucatan sites were estimated by R.B., and for trees at the Oaxaca site by both P.G. and R.B. Note that these estimates are only gross indications for differences in light levels between sites and that actual light levels for trees in the dry site are possibly higher in the same CII class compared with the moist site as crown structure in the dry site is more open. Tree heights were mostly estimated by eye by R.B. and P.G. and calibrated with reference to measurements from felled trees.

### Ring width and isotope measurements

Samples were air-dried and sanded until rings were visible, and measured using a ring measurement device (Velmex or LINTAB). Rings width measurements from different radii, or cores were averaged and converted to diameter growth (see Brienen and Zuidema 2005). Carbon isotope ratios of the tree ring cellulose (δ^13^C_tr_) were measured over the trees’ entire lifetimes for a subset of 28 large trees, and over the last 2–10 rings for 72 smaller trees to assess effects of tree size and crown illumination, totaling more than 3200 δ^13^C_tr_ measurements. For the site in Yucatan, only large trees were analyzed for δ^13^C_tr_. The oldest ages of trees included in this analysis are 110 years for the Yucatan site, 118 years for the Oaxaca and 203 years for northern Bolivia.

Individual rings were cut using a scalpel and cellulose was extracted following the batch method of Wieloch et al. (2011). Cellulose was homogenized and then freeze-dried, and weighed in tin capsules. Isotope analysis was undertaken at the German Research Centre for Geosciences (GFZ, Postdam and Julich, Germany) for samples from Bolivia and Yucatan, at the National Environmental Isotope Facility (NEIF), British Geological Survey, for samples from Bolivia, Selva Negra and at the University of Leicester (UK) for samples from Oaxaca.

We calculated Δ^13^C_tr_ according to Eq. (1), and using atmospheric records of δ^13^C_a_ obtained from Antarctic ice cores (Francey et al. 1999), complemented with recent data from Mauna Loa from the NOAA ESRL Global Monitoring Laboratory (http://www.esrl.noaa.gov/gmd/ccgg/trends/full.html). We did not calculate internal leaf CO_2_ concentrations (*c*_i_) or derive intrinsic water-use efficiency (iWUE), as different equations have been used including models with uncertain terms for mesophyll conductance and photorespiration (Seibt et al. 2008, Schubert and Jahren 2015) causing unnecessary complexity and uncertainty in the interpretations of plant isotope discrimination for our purpose. We did however correct all our calculations of Δ^13^C_tr_ for lower δ^13^C_a_ above the soil due to respiration of depleted soil organic carbon (Buchmann et al. 1997). This was done using a previously developed relationship for the difference between below- and above- canopy δ^13^C_a_ and tree height using literature data for tropical forests (see Brienen et al. 2017). These adjustments were relatively small for trees taller than 3 m (84% of all data) (~0.1‰ decrease in Δ^13^C_tr_), but larger (2–3.5‰) for a small portion of rings formed when trees were <1 m in height.

### Analyzing lifetime change in Δ^13^C

To assess controls of lifetime change in Δ^13^C, we first related Δ^13^C to age, tree height, diameter growth and atmospheric CO_2_ (*c*_a_) for large trees with long trajectories containing more than 40 years of data. For each site, we selected the most parsimonious model to explain variation in Δ^13^C based on r-squared and AIC selection criteria. Separate analysis was performed using the Δ^13^C records of the last five rings, including a range of different tree sizes to additionally test for the influence of crown illumination on Δ^13^C.

We assessed if Δ^13^C differed between periods of suppressed versus high growth, and if Δ^13^C changed during growth releases. Suppressions were defined as periods of growth lower than a defined threshold over at least five consecutive years. Thresholds were calculated as the midpoint of mean growth at CII2 and CII3 (see Figure 3). Growth releases were defined as percent growth changes greater than 100% between two adjacent 10 year windows (see Brienen et al. 2010*a*).

### Growth–Δ^13^C relationships

To compare changes in growth–Δ^13^C relationships across life stages, we defined a size threshold to distinguish between understory and canopy trees. Canopy trees were defined as those taller than about two-thirds of the total local forest canopy. We chose this threshold as more than 80% of the trees above these heights had their crowns exposed to full overhead light (i.e., reached a CII index of 3b or higher). Thresholds corresponded to heights of 7.5, 10 and 17 m and diameters of 15, 20 and 30 cm for Oaxaca, Yucatan and Bolivia, respectively (Table 1, Figure 1).

Growth–Δ^13^C relationships were analyzed using a simple Pearson’s *r* correlation analysis on two types of data: (i) raw data and (ii) detrended (i.e., high-frequency) data from which we removed effects arising from age or ontogenetic effects and from canopy gap dynamics to enhance the underlying climate signal (Cook and Peters 1980). The detrended data were calculated for both ring width and Δ^13^C as the ratio of raw values to smoothing trends using a flexible spline with a rigidity of 15 years and a wavelength cut-off of 0.8. Examples of these splines are shown in Figure 4 and Figure S2, available as Supplementary data at *Tree Physiology* Online.

Pearson correlation analyses were performed in the following three different ways:

(i) All-data correlations using and mixing all data from different trees and years. Correlations in this analysis are due to variation in growth and Δ^13^C within trees as well as between trees. This analysis was done for understory and canopy stages as well as for small size classes of 5-cm widths using raw and detrended data.
(ii) Within-tree correlations were calculated as the mean of correlations between growth and Δ^13^C within individual trees using both raw and detrended data. These correlations were calculated for understory and canopy stages, for trees with more than 40 years of data.
(iii) Between-tree correlations were calculated between mean growth and Δ^13^C of different trees for canopy and understory life-stages, as well as for small size classes of 5-cm widths.

These different types of analyses provide different insights: within-tree correlations highlight temporal co-variation between growth and Δ^13^C, while the between-tree correlations highlight variation between individuals due spatial differences in, e.g., soil conditions or light availability, and all-data correlations capture both effects.

## Results

### Lifetime changes in carbon isotope discrimination (Δ^13^C)

**Figure 2.**
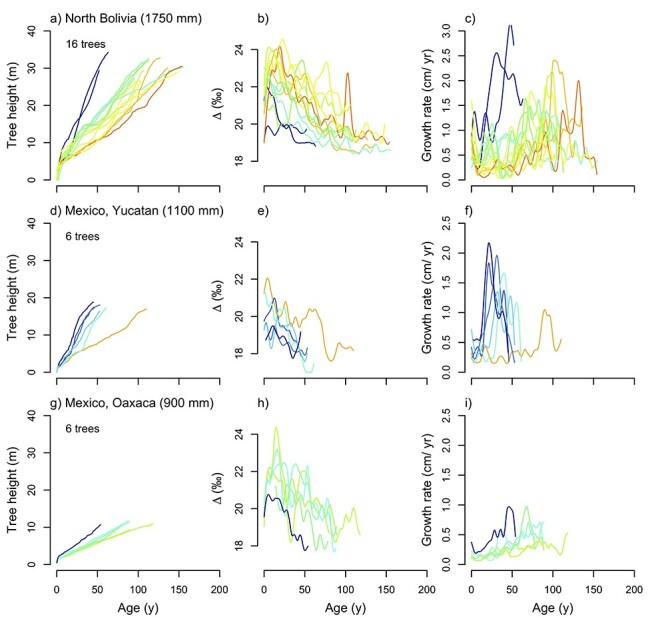
Relationships of tree height, C-isotope discrimination (Δ^13^C) and growth with tree age. The color scheme in figures corresponds to the trees’ average growth rates in understory, defined as trees smaller than 30, 20 and 15 cm in diameter for Bolivia, Yucatan and Oaxaca, respectively (see Materials and methods). Tree heights in left panels were estimated from tree diameter and site-specific mean diameter-height allometries (see Figure 1b). For clarity, curves for Δ^13^C and growth are smoothed using smoothing splines (see Materials and methods).

At all three sites, carbon isotope discrimination (Δ^13^C) decreased strongly over a tree’s lifetime from maxima of 24–25‰ in the understory to minima of 17–18‰ in the canopy stage (Figure 2). Tree height is the strongest predictor for lifetime changes in Δ^13^C in Bolivia, explaining more variation than age, growth or [CO_2_]. For the other two sites, height and [CO_2_] are equally strong predictors for changes in Δ^13^C (Table 2). The change in Δ^13^C with tree height was greatest in the driest site in Oaxaca (−0.41 ± 0.02‰ m^−1^), intermediate for Yucatan (−0.20 ± 0.01‰ m^−1^) and smallest for Bolivia (−0.15 ± 0.00‰ m^−1^). Within individual trees, we found a close relationship between lifetime changes in Δ^13^C and tree height, with tree height explaining 50–86% of the variation in Δ^13^C in most trees (Figure S1, available as Supplementary data at *Tree Physiology* Online). Variation in Δ^13^C between and within trees was generally greatest in the understory stage and was especially large in the wettest site in Bolivia (Figure 2).

**Table 2:** Individual effects of tree height, growth, crown illumination, age and [CO_2_] on Δ^13^C; values shown are the R^2^; significant relationships are shown in bold; note that all relationships here are negative.

Site	Height	Diameter growth	Basal area growth	Crown illumination	Age	[CO_2_]	Most parsimonious model^2^	R^2^
Lifetime change in Δ^13^C								
Northern Bolivia	**0.59**	**0.18**	**0.26**	NA	**0.43**	**0.33**	Height, Diameter growth	**0.66**
Yucatan	**0.45**	**0.07**	**0.19**	NA	**0.20**	**0.46**	Height, Diameter growth	**0.48**
Oaxaca	**0.31**	**0.18**	**0.23**	NA	**0.25**	**0.38**	[CO_2_], Diameter growth	**0.41**
Last 5-years^1^								
Northern Bolivia	**0.75**	**0.12**	**0.41**	**0.52**	**0.62**	−	Height, Crown illumination	**0.77**
Oaxaca	**0.55**	0.15	**0.41**	**0.81**	**0.41**	−	Crown illumination	**0.81**

^1^No last 5-year data across diameter or light classes available for Yucatan.

^2^From a comparison of models with diameter growth and either Height, CO_2_ or Age. Due to strong collinearity, only one of these variables was included. The most parsimonious model was chosen based on highest R^2^ and lowest AIC.

### Effects of light on growth and carbon isotope discrimination (Δ^13^C)

**Figure 3.**
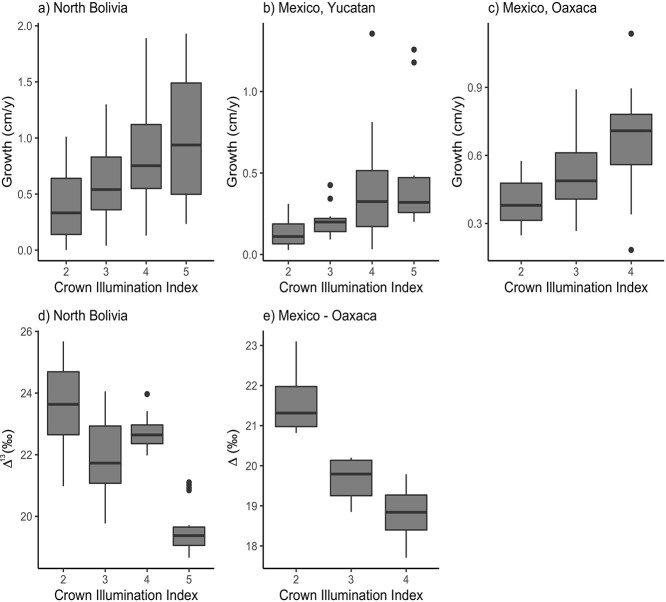
Diameter growth rate and isotope discrimination (Δ^13^C_tr_) in relation to crown illumination index (CII). The CII varies from 2 for sapling and seedlings without direct sunlight to 4 and 5 for trees with full direct sunlight from above or for emergent crowns. Both growth and isotope discrimination are calculated as the mean over the past five rings for extant trees. Boxes denote 25th, 50th and 75th percentile and whiskers extend to the largest or smallest value no more than 1.5 times the interquartile range. Letters indicate significant (*P* < 0.05) differences between CII classes using paired *t*-tests. In all three sites, CII explains greater amount of variation than diameter using ANOVA with tree diameter as covariable.

Variation in Δ^13^C of the five most recent rings of extant trees was most strongly related to tree height and light (i.e., CII) at the two sites for which we had data (Table 2, lower panel). An additional relationship between Δ^13^C and age arose mainly due to the correlation between age and tree height but disappeared in a multiple regression when accounting for their co-linearity. Trees with limited direct sunlight (i.e., low CII) had significantly lower growth and higher Δ^13^C (Figure 3). This effect of CII may partially arise due to the correlation between tree height and higher CIIs. Our analysis showed however that the effects of CII on growth only disappeared in Oaxaca after correcting for tree height, and the effects of CII on Δ^13^C remained significant at both sites. Note that full statistical separation of the strongly correlated effects of height and CII on Δ^13^C and growth is difficult.

### Relationship between growth and carbon isotope discrimination (Δ^13^C)

**Figure 4.**
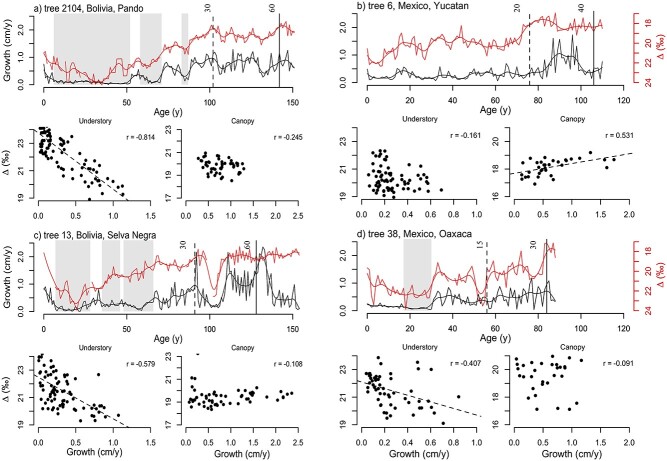
Examples of trajectories for growth (black lines) and C-isotope discrimination (Δ^13^C, red lines in reversed axes) of individual trees, and scatterplots of growth versus Δ^13^C for understory and canopy growth phases. Note the reverse axis for Δ^13^C time series in red in the upper panels. Understory is defined as trees smaller than 30, 20 and 15 cm in diameter for Bolivia, Yucatan and Oaxaca, respectively, which is the approximate size at which trees reach the lower canopy (see Materials and methods). The age at which the individual trees reached these diameter thresholds for understory versus canopy growth phases or larger canopy sizes are indicated in the trajectory plots with vertical broken and continuous lines, respectively, with numbers referring to tree diameter in cm. The trend lines in the scatter plots indicate significant (*P* < 0.05) relationships between early growth rate and Δ^13^C, and Pearson correlation coefficients (*r*) are shown. Smooth curves for growth and Δ^13^C trajectories are smoothing splines (see Materials and methods). Periods of suppression (i.e., minimum of 5 years of growth below a site-specific threshold, see Materials and methods) are indicated by shaded areas.

Figure 4 shows some chosen examples of lifetime growth and Δ^13^C trajectories for individual trees. These examples as well as most trees (see Figure S2, available as Supplementary data at *Tree Physiology* Online, for all individual trajectories) illustrate strong temporal changes in growth especially for trees in the moist site in Bolivia going through prolonged periods of slow and fast growth when in the understory (Figure 4a and c). The shaded areas indicate periods of growth suppression. These periods of suppressed growth were generally associated with higher Δ^13^C (note the reverse axis for Δ^13^C). Differences in Δ^13^C between periods of suppressed and non-suppressed growth in the understory trees were significant for Bolivia (Δ^13^C_suppression_ = 22.6‰, Δ^13^C_high_growth_ = 21.3‰, *P* < 0.001) and Oaxaca (Δ^13^C_suppression_ = 21.8‰, Δ^13^C_high_growth_ = 20.7‰, *P* < 0.001), but not for Yucatan (Δ^13^C_suppression_ = 19.5‰, Δ^13^C_high_growth_ 19.7‰, *P* = 0.6). Across all trees, we further found that growth releases (i.e., growth increases >100% between two subsequent 10-year periods) were associated with a 0.70‰ decrease in Δ^13^C (Welch two sample *t*-test, *t* = 7.4, *P* < 0.001).

The scatterplots in Figure 4 show the relationship between growth and Δ^13^C for each tree in both the understory (left panel) and canopy stages (right panel). The two examples for Bolivia clearly show strong negative relationships between Δ^13^C and growth when trees were in the understory (i.e., when <30 cm in diameter), but correlations disappeared after trees reached the canopy (>30 cm in diameter). The example for Oaxaca shows a similar pattern, while the Yucatan example shows no correlation in the understory stages and positive correlations between growth and Δ^13^C after reaching the canopy.

### Growth–Δ^13^C relationships for understory versus canopy trees

**Figure 5.**
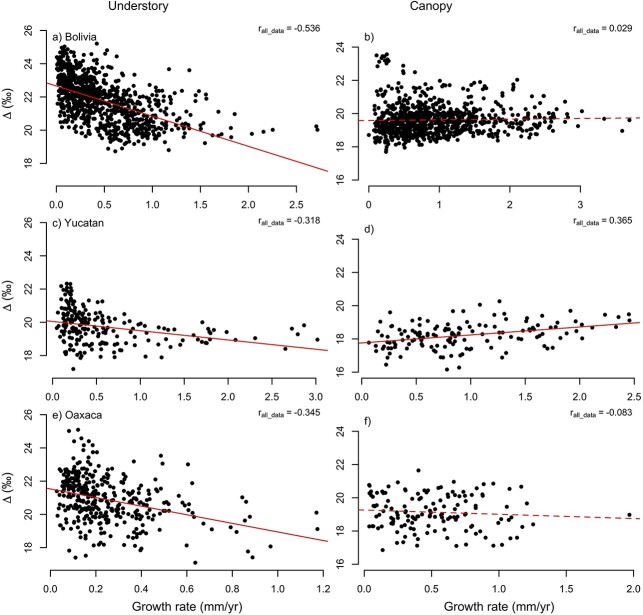
Relationship between discrimination and diameter growth using all data from all trees, separated between understory and canopy stages, per site. Correlation coefficients (*r*) are shown. Continuous lines indicate significant relationships (*P* < 0.05), and broken lines non-significant relationships (*P* > 0.05). See Figure S3, available as Supplementary data at *Tree Physiology* Online, for the same analysis using detrended data.

We first assessed changes in growth–Δ^13^C relationships between different life-stages by separating data into understory and canopy stages. This showed that during the understory stage, growth and Δ^13^C were negatively related, but there was no relation (Bolivia and Oaxaca), or a weakly positive relationship (Yucatán) at the canopy stages (Figure 5). This analysis mixes data from different trees and multiple years within trees, and relationships may thus arise from between-tree as well as within-tree co-variation between growth and Δ^13^C (e.g., examples in Figure 4). Further analyses showed that both factors cause anti-correlations in the understory stages in Bolivia with relatively strong mean within-tree correlations (*r* = −0.50) as well as between-tree correlations (*r* = −0.70, Table 3). In contrast, for the two other sites within-tree correlations in the understory were weaker (*r* = −0.18 and −0.35), but between-tree correlations are relatively strong (*r* = −0.59 and −0.57). At the canopy stages, most correlations disappeared apart from within-tree correlations in Yucatan, which change to positive correlations. We also found some strong negative between-tree correlations for canopy trees at the site of Oaxaca, but sample sizes were very low (Table 3).

**Table 3:** Correlation coefficients between growth and Δ^13^C for understory and canopy trees for the three sites; within tree correlations are calculated as the mean of individual tree correlations that have at least eight data points using raw and detrended data; correlations between trees consist of correlations between the mean growth and mean Δ^13^C in the understory and canopy life stages; understory is defined as trees smaller than 30, 20 and 15 cm in diameter for Bolivia, Yucatan and Oaxaca, respectively; values between brackets for the within-tree correlations give the standard error of the variation between trees in correlation coefficients. ns, ^*^, P< 0.05.

	Mean correlation (*r*) within trees				Correlation (*r*) between trees		
	Raw data		Detrended data		Raw data		
	Understory	Canopy	Understory	Canopy	Understory	Canopy
Bolivia	−0.50 (0.08)	−0.07 (0.08)	−0.11 (0.06)	−0.01 (0.06)	−0.70^*^	0.09^ns^
Yucatan	−0.18 (0.14)	0.41 (0.09)	0.06 (0.10)	0.43 (0.07)	−0.59^ns^	0.19^ns^
Oaxaca	−0.35 (0.4)	0.18 (0.13)	0.00 (0.08)	0.19 (0.10)	−0.57^ns^	−0.71^ns^

**Figure 6.**
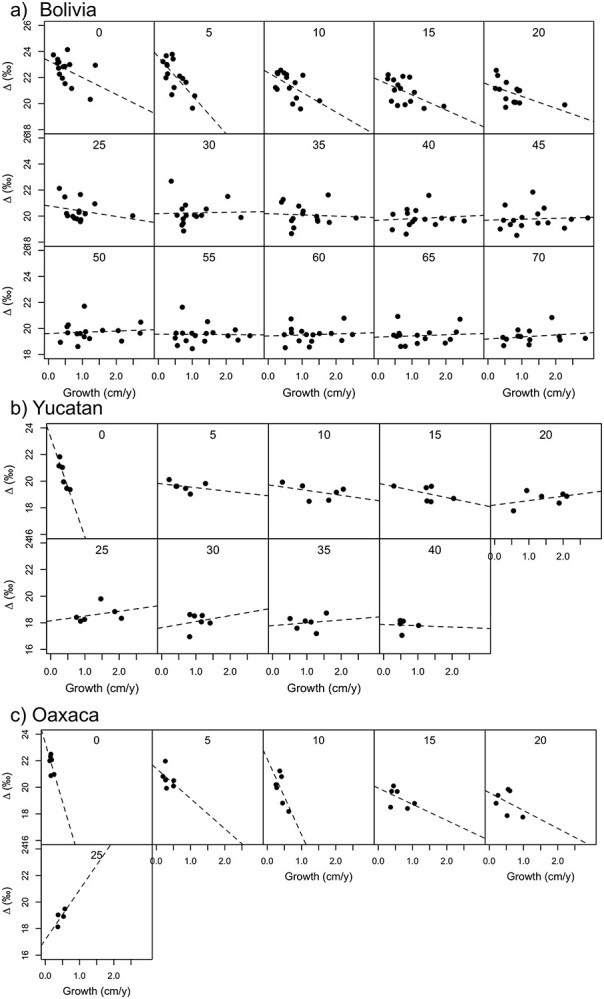
Relationship between mean diameter growth and Δ^13^C in diameter classes of 5-cm widths for Bolivia (a), Yucatan (b) and Oaxaca (c). Each point represents the mean of diameter growth and Δ^13^C for a different tree. Numbers at the tops of the graphs denote the lower boundary for each size class in cm.

**Figure 7.**
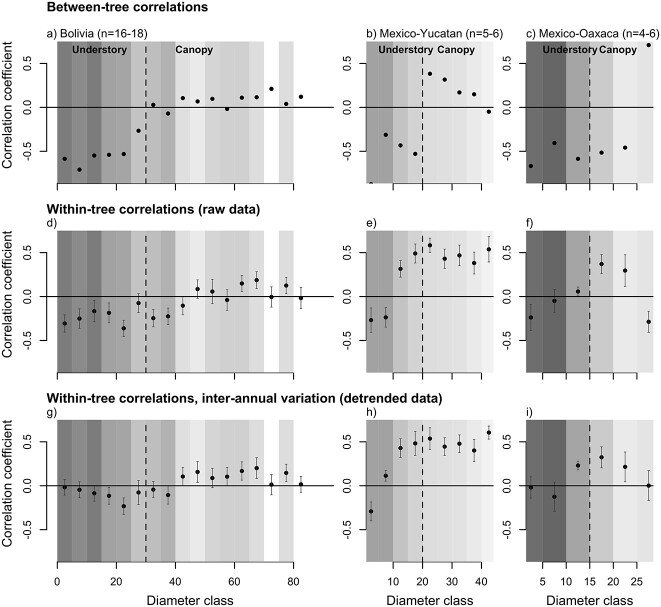
Correlation coefficients between growth and discrimination by diameter class for three sites between trees (cf., Figure 6) and within-trees using detrended data. Vertical broken lines indicate the approximate size at which trees reach the canopy for the three sites, respectively, at 30, 20 and 15 cm in diameter for Bolivia, Yucatan and Oaxaca. The gray scale of the background corresponds to the average crown exposure index, with darker colors representing lower light levels. Error bars in panels (d–i) indicate the standard error of the variation between trees in correlation coefficients (i.e., standard deviation/sqrt(n)). Sample sizes refer to the number of trees included in the correlations for each size class.

We further assessed changes in mean between-tree growth-Δ^13^C_tr_ relationships of trees across size classes (Figures 6 and Figures 7a-c). This analysis showed strong negative relationships between mean growth and Δ^13^C in the smallest size class (0–5 cm diameter) at all three sites. In Bolivia, the slope of the negative relationship weakened gradually and completely disappeared from ca 30 cm DBH onward, while in Yucatan, the relationship deteriorated immediately after the smallest size class. In Oaxaca, negative relationship remained apparent until 25 cm diameter.

### Effects of inter-annual variation in growth and Δ^13^C

To assess the effects of year-to-year variation in climate on growth and Δ^13^C, we used detrended data that removed variation due to ontogeny and gap dynamics. This analysis showed no or weak correlations between detrended growth and Δ^13^C in the understory stage for all three sites, but strong positive correlations at the canopy stage for the site of Yucatán (Table 3). At this site, positive relationships between detrended growth and Δ^13^C were observed from relatively small diameters onward (i.e., >10 cm DBH, Figure 7h). In Bolivia, positive relationships between detrended growth and Δ^13^C were only evident for trees larger than 50-cm DBH (Figure 7g). At the site of Oaxaca, we found positive relationships between detrended growth and Δ^13^C at intermediate sizes of 10–20 cm diameter (Figure 7i).

## Discussion

### Lifetime changes in Δ^13^C

Isotope discrimination (Δ^13^C_tr_) decreased over tree’s lifetimes by 4–6‰ in all three sites. These decreases are not due to below-canopy profiles of δ^13^C_air_ or plant responses to changing atmospheric CO_2_ but are caused by height-related changes in microclimate (e.g., light, humidity and temperature) and in tree structure and functioning as trees grow through the canopy. Below-canopy ^13^C depletion due to soil respiration (cf., Buchmann et al. 1997) does not explain the observed Δ^13^C trends as all values of Δ^13^C were ‘adjusted’ for any impact from the respiration of soil carbon (see Materials and methods), plus the impact is estimated to be relatively small (2–3‰) and affects the first few rings only. The trends in Δ^13^C are also not due to long-term CO_2_ increases. Firstly, our results show that the effect of CO_2_ is weaker than height-related effects (Table 2). Secondly, the most common plant response to increasing CO_2_ is either to maintain a constant Δ^13^C (i.e., constant *c*_i/_*c*_a_ ratio, Saurer et al. 2004, Franks et al. 2013, van der Sleen et al. 2015) or to increase Δ^13^C (Voelker et al. 2016, Keeling et al. 2017), while we find strong decreases in Δ^13^C over time (i.e., tree age).

The observed decreases in Δ^13^C and thus leaf intercellular CO_2_ (*c*_i_) with tree height are in line with temperate forest trees (e.g., mean of 6‰ in McDowell et al. 2011, 0–6‰ in Brienen et al. 2017, 1.2–4.5‰ in Klesse et al. 2018, 3.7–7.2‰ in Vadeboncoeur et al. 2020) and are thus due to greater increases in CO_2_ demand for assimilation relative to stomatal conductance (CO_2_ supply). As trees increase in height, stomatal conductance (*g*_s_) becomes more rate limiting for CO_2_ uptake, due to stomatal closure in response to increases in leaf temperature (Fauset et al. 2018) and thus VPD associated with higher irradiance (Lloyd and Farquhar 2008), as well as increases in hydraulic pathlength and resistance resulting in decreases in leaf water potentials (Koch et al. 2004, Ryan et al. 2006). The relative contributions of the limitation through hydraulics (i.e., water transport to the canopy) versus changes in canopy level irradiance and VPD in controlling these height-related changes in Δ^13^C are still debated (McDowell et al. 2011, Vadeboncoeur et al. 2020), but our results suggest a dominant influence of irradiance. Firstly, we find that the difference in discrimination between small understory and canopy trees (Figure 2b) is of similar magnitude to the difference between sun and shade trees (Figure 3). Secondly, the absolute magnitude of lifetime change in discrimination (from understory to canopy trees) does not vary between sites, despite the large difference in maximum tree height between sites, pointing perhaps toward a relative limited role of hydraulic limitation in C isotope discrimination. Thirdly, we find that once trees reached the canopy, Δ^13^C remained relatively constant or showed only slight decreases, especially at the Bolivian sites (Figure 2b, Figure S1, available as Supplementary data at *Tree Physiology* Online). Thus, light seems one of the most critical drivers for variation in Δ^13^C in line with previous studies (McDowell et al. 2011, Brienen et al. 2017, Klesse et al. 2018, Vadeboncoeur et al. 2020). Apart from direct effects of light on discrimination, leaf morphological changes, such as leaf thickness (Aranda et al. 2007), leaf nitrogen (Duursma and Marshall 2006) and leaf mesophyll conductance (Seibt et al. 2008), have also been shown to affect discrimination. Leaf morphological changes between sun and shade leaves in the two *Cedrela* species are large with, for example, an almost twofold variation in a specific leaf area (Poorter and Hayashida-Oliver 2000, Guzmán-Q et al. 2016). Regardless of the causes, lifetime trends in Δ^13^C from understory to canopy show increasing limitations of stomatal conductance on carbon assimilation, most likely driven by a combination of increased evaporative demand (irradiance) and restrictions to water supply (hydraulic limitations). The extent of the relative contributions from these drivers still requires further study.

Finally, despite large differences in mean annual rainfall (900–1750 mm), Δ^13^C does not vary strongly between sites and converges to a narrow range (~18–20‰) once trees reached the canopy, contrasting with general observations of increases in discrimination with rainfall (Schulze et al. 2006, Prentice et al. 2011, Givnish et al. 2014). This may be due to the relatively small differences in water availability during the rainy season, the actual growing period for this deciduous species. Alternatively, decreases in soil water potential across sites could be compensated for by decreasing maximum tree heights toward drier sites (Figure 1), or hydraulic adjustments such as the ratio of leaf area to water-conducting tissue could result in maintenance of similar leaf water potentials (Mencuccini and Grace 1995, McDowell et al. 2002, McDowell et al. 2011), and thus Δ^13^C, for canopy trees.

### Co-variation of Δ^13^C and growth in the understory

Previous analysis of ring width data alone suggested that the observed growth rate variation in the moist forest in northern Bolivia was due to variation in light related to canopy gap dynamics (Brienen and Zuidema 2005). Here, we confirm this interpretation using the paired ring width–Δ^13^C approach. We find a strong match in temporal variation between Δ^13^C and growth in the understory within individual trees (Figure 4a and c, Table 3). Periods of low growth (i.e., suppressions) were associated with higher Δ^13^C, while growth increases (i.e., releases) led to decreases in Δ^13^C. Increases in Δ^13^C and *c*_i_ during periods of low growth indicate that growth is limited by demand for CO_2_, and not by CO_2_ supply through stomatal conductance (which would result in decreases in *c*_i_). Theoretically, the negative covariation between growth and Δ^13^C could be due to variation in nutrients (Cernusak et al. 2013), but nutrients are unlikely to vary over time and cause temporal covariation of growth and Δ^13^C within trees. In all, these results provide strong evidence that growth in the understory is light limited, consistent with the observed effect of crown illumination on Δ^13^C and growth (Figure 3). However, we also find large variation in Δ^13^C at low growth rates (Figure 5a), indicating that growth of understory trees may not be exclusively limited by light and that water stress could play a role by reducing stomatal conductance and growth in some years.

Consistent with the temporal co-variation of growth and Δ^13^C within trees, we find that mean growth and Δ^13^C are also strongly negatively correlated between small trees in Bolivia (Figure 6a). Fast-growing, small trees had lower average Δ^13^C than slow-growing trees, resulting in strong negative relationships between growth and Δ^13^C in the smallest size classes. The negative slope of these relationships gradually weakened toward larger size classes, indicating diminishing controls of light on variation in growth and/or Δ^13^C with increasing tree size. The size at which this relationship disappeared in Bolivia is surprisingly close to the estimated size at which most trees reach (sub)canopy levels and receive full overhead light (~30 cm diameter).

At the two drier sites, we also observed negative correlations between growth and Δ^13^C in the understory, both within trees, as well as between trees (cf., Table 3, Figure 6). At the Yucatan site, the strength of the correlations declines rapidly as trees get bigger, showing that light is only a limiting factor to growth in the smallest trees (<5 cm). This can be explained by the canopy being lower and more open, with trees at this site showing faster canopy accession and significantly shorter periods of suppressed growth compared with the moist site (Brienen et al. 2010*a*). Patterns are different for the site of Oaxaca as negative relationships between growth and Δ^13^C continued even for large trees that had reached canopy positions with full sunlight (Figure 6c). These negative growth–Δ^13^C relationships for canopy trees are entirely due to differences in growth and Δ^13^C between trees (Figure 6c), as within-tree correlations are slightly positive (Table 3). As this site had a very open and low canopy structure, light was not expected to be a limiting growth factor for taller trees. Besides light, spatial variation in soil fertility could be driving variation in growth and Δ^13^C.

In all, paired growth–Δ^13^C showed that spatial and temporal variation in light limits growth at all three sites during the understory life phases. At all three sites, those fast-growing trees that reached the canopy early had the lowest mean Δ^13^C (cf., dark colored trajectories in Figure 2), indicating that light is the main control behind growth differences. Sites differed in the strength and the duration of light limits on growth, with the strongest limitations at the moist site with the highest and most dense canopy structure.

### Interannual variation in growth and Δ^13^C

Detrended growth and Δ^13^C showed positive correlations at inter-annual scales in Yucatan once trees reached diameters >10 cm, and some weaker positive correlations for mid-sized and large trees in Bolivia and Oaxaca (Figure 7g–i). Positive correlations are indicative for water stress reducing growth in dry years through greater reductions in stomatal conductance, resulting in lower leaf intercellular CO_2_ (*c*_i_) and lower Δ^13^C, caused by either reduced soil water content or leaf water status controlled by VPD (Tardieu and Davies 1993, Harris et al. 2004). The differences in *C. odorata* between Yucatan and Bolivia are consistent with other studies showing strong positive relationships between growth and Δ^13^C at semi-arid sites (Andreu et al. 2008, Brienen et al. 2011, Leavitt et al. 2011, Voelker et al. 2014), and a weakening or even opposite directions of correlations at sites with greater water availability (Brooks et al. 1998, Young et al. 2010, Voelker et al. 2014).

The lack of correlations between inter-annual variation in growth and discrimination for trees at Oaxaca is surprising as it is the driest site and as inter-annual variation in growth by itself shows relatively good synchrony between trees (Groenendijk 2010). These results thus probably do not reflect insensitivity to climate but may be due to decoupling of leaf level isotope discrimination and growth as a result of post-photosynthetic fractionation processes and use of stored carbohydrate reserves (Gessler et al. 2014), or asynchronism between canopy photosynthesis and actual cambial activity and wood growth (Wagner et al. 2016).

The increase in strength of the association between growth and Δ^13^C_tr_ as trees get bigger in Yucatan (and to lesser degree in Bolivia) is consistent with observations (Mérian and Lebourgeois 2011, Bennett et al. 2015, Trouillier et al. 2019, McGregor et al. 2021) and theory (Ryan and Yoder 1997) of strengthening climate-growth correlations with increased tree size. Taller trees have a longer hydraulic pathlength, and larger evaporative demand, which increase trees’ water stress (McDowell and Allen 2015). Our results indicate that any potential increase in rooting depth and soil water uptake as trees grow bigger (Dawson 1996, Brum et al. 2019) is not sufficient to completely offset increased water demand for trees in the canopy of *Cedrela*, which is consistent with its shallow root system and reliance on water from the top soil (Kunert et al. 2010). A greater control of water availability on growth variation of canopy trees in Yucatan could be indicative of greater mortality risk under drought, as shown recently (DeSoto et al. 2020), and explain significantly shorter lifespan (Figure 2) and tree height (Figure 1) in this dry site compared with the wetter forest in Bolivia.

**Figure 8.**
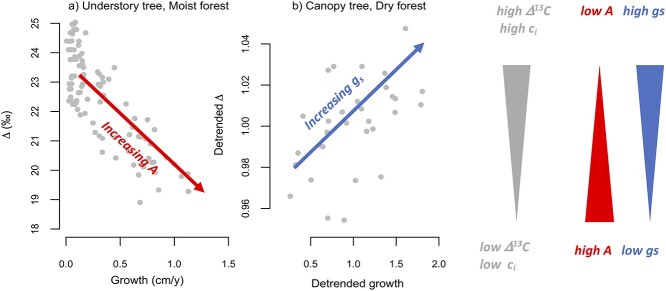
Graphical summary of opposing growth–Δ^13^C correlations of *Cedrela* trees in understory (Bolivia) and canopy phases (Yucatan) indicating light (a) and water (b) limitations on growth. Increasing discrimination results from increase in *c*_i_/*c*_a_, and can be caused by increases in stomatal conductance (*g*_s_, relative to *A*) or decreases in assimilation rate (*A*, relative to *g*_s_). Increasing growth combined with decreasing discrimination (panel a) indicates a release of constraints of photosynthetic carboxylation rate on growth (i.e., greater *A* due to increase in light), whereas increasing growth with increase in discrimination indicates increasing stomatal conductance as the principal driver for increased growth (panel b).

In summary, we observed a distinct change in drivers of growth rate variation from light limitation in the understory to drought limitations in the canopy. This is especially pronounced when comparing correlations between growth and discrimination for small trees in the moist site with large trees of the same species in the dry site (see Figure 8). Opposite directions of correlations indicate different fundamental controls on growth rates at different life stages and sites.

### Further implications for tree ring studies

We find an increase in the strength of the co-variation between growth and Δ^13^C as trees reach the canopy, especially in Yucatan. This may be due to either true changes in climate sensitivity of trees (i.e., magnitude of the response in growth or Δ^13^C per change in climate variable) or simply to reductions of other influencing factors as trees get bigger (e.g., light). Whatever the causes, these size- or age-related changes in variance caused by climate are common across many species (Carrer and Urbinati 2004, Mérian and Lebourgeois 2011, Trouillier et al. 2019). This may violate the stationarity principle for growth–climate relationships in dendroclimatic reconstructions (Wilmking et al. 2020) and affect assessment of (changes in) trees’ drought sensitivity over time (Anderegg et al. 2020) or in response to CO_2_ (Zuidema et al. 2020). This highlights that ring width variation in the juvenile phases is unlikely to provide a reliable climate proxy, especially for shade-tolerant species, and disentangling ontogenetic effects from climate or CO_2_-related effects requires great care.

Our results also have significant implications for the use of δ^13^C_tr_ to assess responses of iWUE of trees to climate or atmospheric CO_2_. Understory trajectories that are governed by light, as observed here, cannot be interpreted meaningfully in such a context. In our case, these understory phases extended to more than 100 years in at least one tree in the wettest site (cf., Figure 3a), which is significantly longer than the few decades of ‘juvenile’ phase suggested to be excluded (Gagen et al. 2008). This may vary however between species and sites; according to our criteria (see Materials and methods), the average age when reaching the canopy ranged for *C. odorata* from 37 years in Yucatan to 64 years in Bolivia. Similarly, shade-tolerant species may have much stronger and longer-lasting trends (Vadeboncoeur et al. 2020) compared with shade-intolerant tree species, which do not survive long in the shadow and may lack longer term trends (McCarroll et al. 2020). Even a lack of trends in isotope series (McCarroll et al. 2020) need to be interpreted cautiously, however, as insidious trends due to ontogeny can be hard to distinguish from climate or CO_2_ effects (Brienen et al. 2017). The danger of possible mis-interpretations of ontogenetic trends in Δ^13^C for trees’ responses to CO_2_ is real. For example, Adams et al. (2019) recently showed that increases in iWUE were two times larger in dry compared with wetter tropical forests, but these differences are of the same magnitude as observed in this study, which we show to arise due to differences in the canopy height and structure between forests (Figure 2). Results from Adams et al. (2019) may thus similarly be due to variation in tree height and leaf area index with water availability (Klein et al. 2015, Tao et al. 2016), rather than to different responses to CO_2_.

## Conclusions

We showed that paired measurement of ring width and Δ^13^C can provide powerful insights in the causes of growth rate variation over trees’ lives. This approach could be highly effective for studying gap regeneration in closed canopy forests (Canham 1989, Clark and Clark 2001) and provide insights in the role of large-scale disturbances in tropical forest dynamics (Vlam et al. 2017) or the causes of tree death by allowing distinguishing light and drought effects on, e.g., pre-death growth declines (Cailleret et al. 2017, Gessler et al. 2018). We find significant shifts in controls on growth over a tree’s lifetime, from light availability when trees are small to drought when trees are tall, with light limitations playing a much more pronounced role in moist forests compared with dry forests. Two lines of evidence suggest increasing limitations of stomatal conductance on carbon gains as trees increase in height and reach full canopy positions. Firstly, at all three sites, we find monotonic decreases in discrimination with increasing tree height, and thus leaf internal CO_2_ concentration (*c*_i_), indicating that stomatal conductance increasingly becomes the bottleneck for carbon assimilation. Secondly, we find that interannual variation in growth and discrimination are increasingly positively correlated when reaching larger tree heights, especially at one of the drier sites. These results are consistent with hydraulic limitation theory, as described by Ryan et al. (2006), and indicate increasing limitations of stomatal conductance on growth for canopy trees. Trees are not limited by one single resource, but resource limitations may change dramatically over a tree’s lifetime. Analysis of ring widths and carbon isotope ratios, as applied here, provides a powerful tool to partition between environmental factors controlling growth over a tree’s life.

## Supplementary Material

Brienen_et_al_2021_Supplementary_information_tpab142

## Data Availability

All the data and codes used in this publication are available from the authors upon request.
