# Supplementary material for: Paired analysis of tree ring width and carbon isotopes indicates when controls on tropical tree growth change from light to water limitations
Source: Tree Physiol. 2021 Oct 29;42(6):1131–48. doi: 10.1093/treephys/tpab142 (PMC9190751; doi:10.1093/treephys/tpab142)
Supplement: Brienen_et_al_2021_Supplementary_information_tpab142 [file brienen_et_al_2021_supplementary_information_tpab142.docx]

# Supplementary information

**From light to water limitations: carbon isotopes indicate changing controls on tropical tree growth as trees reach the canopy**

Roel Brienen^1^, Gerard Helle^2^, Thijs Pons^3^, Arnoud Boom^4^, Manuel Gloor^1^, Peter Groenendijk^5,6^, Santiago Clerici^1^, Melanie Leng^7^, Christopher Jones^1^

^1^ School of Geography, University of Leeds, Leeds LS6 9JT, UK

^2^GFZ — German Research Centre for Geosciences, Section 5.2 Climate Dynamics and Landscape Evolution, Telegrafenberg, 14473 Potsdam, Germany

^3^Plant Ecophysiology, Institute of Environmental Biology, Utrecht University, 3512 PN Utrecht, the Netherlands

^4^ School of Geography, University of Leicester, Leicester LE1 7RH, UK

^5^Department of Plant Biology, Institute of Biology, P.O. Box: 6109, University of Campinas, UNICAMP, 13083-970, Campinas, SP, Brazil.

^6^Ecology and Biodiversity, Institute of Environmental Biology, Utrecht University, Padualaan 8 3584 CH, Utrecht, The Netherlands

^7^National Environmental Isotope Facility, British Geological Survey, Nottingham, NG12 5GG, UK

E-mail for correspondence: r.brienen@leeds.ac.uk

*Version 19 July 2021*

1. Bolivia, Purrisima

1. Bolivia- Selva Negra

1. Mexico, Yucatan

1. Mexico, Oaxaca

Figure S1. Trajectories of discrimination (red line) and tree height (black line) with age for all large trees in four sites. R-squared values correspond to the relations between discrimination and tree height. Vertical broken lines in the trajectory plots indicate the age at which the individual trees reached these size thresholds for understory vs. canopy growth phases.

1. Bolivia, Purissima

b) Bolivia, Selva Negra

c) Mexico- Yucatan

d) Mexico, Oaxaca

Figure S2. Trajectories of discrimination (red line) and diameter growth (black line) with age for all large trees in four sites. Vertical broken lines in the trajectory plots indicate the age at which the individual trees reached these size thresholds for understory vs. canopy growth phases.

SI Fig 3. Relationship between discrimination and growth using all data from all trees, separated between understory and canopy stages for detrended data (see main Fig. 5 for results for raw data). Correlation coefficients (r) are shown. Continuous lines indicate significant relationships (p < 0.05), and broken lines non-significant relationships (p >0.05).
